# Clinical evidence of 595 nm pulse dye laser treatment for viral warts on hands and feet

**DOI:** 10.1111/srt.13460

**Published:** 2023-09-04

**Authors:** Piero Campolmi, Lavinia Quintarelli, Irene Fusco, Lara Ronconi, Tiziano Zingoni

**Affiliations:** ^1^ Section of Dermatology Department of Surgery and Translational Medicine University of Florence Firenze Italy; ^2^ Section of Dermatology Department of Health Sciences University of Florence Firenze Italy; ^3^ El.En. Group Calenzano Italy

**Keywords:** 595 nm laser therapy, hyperkeratotic warts, therapeutic approach, viral warts of hands and feet

## Abstract

**Background:**

Viral warts, induced by infection by different HPV genotypes, are highly frequent in the general population, particularly during infancy and among adolescents. The effectiveness of the 595 nm Pulse dye‐laser in treating viral warts on the hands and feet in both adults and children was investigated in this study.

**Materials and Methods:**

A selection of 203 patients with multiple viral warts was made. All patients underwent 4–5 treatment sessions with a 595 nm pulse Dye Laser (PDL). Treatment outcome was assessed by a quartile scale: 1 indicates no or low results (0%–25% of the lesion area cleared), 2 indicates slight clearance (25%–50% of the lesion area cleared), 3 indicates moderate‐good clearance (50%–75%), and 4 indicates excellent clearance (75%–100%). Patients were asked for a subjective evaluation of the perceived overall results by means of the following score: unsatisfied (1), not very satisfied (2), satisfied (3), and very satisfied (4). Possible side effects were monitored. Results obtained were judged with a photographic evaluation, immediately and at the control visit (6 months after the last laser session).

**Results:**

All patients observed global improvements. Most of the lesions were completely removed after laser therapy. A total of 95% of patients achieved excellent clearance and they were very satisfied following the laser treatment. Relevant side effects were absent in all patients.

**Conclusion:**

PDL treatment with the study device using a wavelength of 595 nm has proven to be a tolerable and safe therapy for viral warts management.

## 1 INTRODUCTION

Viral warts, induced by infection by different HPV genotypes, are highly frequent in the general population, particularly during infancy and among adolescents. According to estimates, 7%−12% of the general population has cutaneous viral warts. Many patients who need primary care also have other concerns, like problems with their external appearance, in addition to pain and discomfort. Although cutaneous viral warts are common, there is no effective cure. Nevertheless, most warts resolve spontaneously, and a large proportion of the remainder respond to simple recommended treatment. Potential treatments must therefore have low side effects and a good risk profile.^1^ The infection might be characterized by a large number of lesions and a recurrent/recalcitrant course in patients with immunosuppressive conditions, such as HIV. The most affected anatomical areas include the palms and soles, periungual regions, and the face. Viral warts required a timely treatment to avoid viral spreading due to autoinoculation. Furthermore, lesions affecting the soles are associated with significant pain.^2^ Treatments include topical agents, such as keratolytic agents (salicylic acid and urea), salicylic acid and lactic acid in flexible collodion, retinoids and 5‐fluorouracil, and physical treatments such as curettage, cryotherapy, electrosurgery, and ablative CO_2_ laser. While topical treatments require a long time to eradicate the infection and closely depends on patient's compliant, physical treatments are associated with a high rate of recurrence and may result in cosmetic issues (e.g., scarring) or irreversible damages (e.g., nail dystrophy).^3^

Salicylic acid is typically regarded as the most frequently prescribed first‐line treatment because many patients opt to self‐administer over‐the‐counter medications before seeing a doctor.^4^

However, some patients, like those with peripheral vascular disease or diabetic peripheral neuropathy, should be advised against using self‐guided, at‐home plantar wart therapy.

For viral warts, some people consider cryotherapy as a second‐line treatment.^5^, ^6^ Other forms of cryotherapy, like dimethyl ether and propane, are accessible without a prescription but show even lower efficacy. Additionally, cryotherapy is linked to more serious side effects than salicylic acid, such as mobility‐restricting pain.^7^, ^8^ Double‐freeze therapy has shown increased efficacy but carries a higher risk of side effects because the lesion is frozen until a 1‐ to 2‐mm ice halo forms, then completely thawed and immediately refrozen.^4^, ^9^ For young children, this therapy is not advised due to the pain associated.^10^

While salicylic acid and cryotherapy are the most commonly used treatments, they are not appropriate for treatment‐resistant warts. For this reason, novel therapeutic approaches are thereby highly welcomed.

Other treatment modalities have been tried with varying degrees of success and little evidence for recalcitrant warts.

The effectiveness and safety of pulse dye laser (PDL) therapy in treating a variety of cutaneous vascular lesions have been extensively documented in the literature.^11^, ^12^

Recently, PDL has been commonly used in the treatment for viral warts and several investigators have been reported that it represents a safe and tolerable therapy with satisfying results.^13^, ^14^, ^15^

According to these scientific findings, the current study presents our experience on the use of 595 nm Pulse dye‐laser combined with salicylic acid ointment (used to provide a reduction of the lesion thickness of hyperkeratosis warts between the different sessions of dye‐laser) for the treatment of hand and feet warts in adults and children.

## 2 MATERIALS AND METHODS

### 2.1 Patients population

For this study 203 patients were treated between 2018 and 2022. The exclusion criteria included photosensitizing drugs, anticoagulants, retinoids, previous exfoliating treatments, surgical treatments, exposure to the sun or UV lamps, and pregnancy. All wart patients were informed of treatment methods including adverse effects. Every patient provided informed consent.

### 2.2 Treatment and study protocol

At the initial visit, all patients were examined carefully to determine the subtype, number, and size of warts. The treatments were carried out as long as the results progressed in accordance with the patient's requirements.

The 595 nm pulse Dye Laser (Synchro VasQ, Deka M.E.L.A, Calenzano [FI], Italy), with fluences of 6–7 J/cm^2^, spot size 12 mm (in cases of small viral warts a spot size of 7 or 10 mm can be used) and pulse duration 0.5−1.5 ms, was used. The treatment was performed at 15–20 days intervals. No anesthesia was used to treat the lesions.

The Salicylic acid ointment, only used in cases of hyperkeratotic warts, was applied the evening before the treatment and then removed before the laser session.

Every laser session included the use of an efficient cooling device, which increased comfort. Patients were advised to use cool gauzes, emollient creams, and sunscreen until full recovery and to avoid the sun and cosmetics in the immediate postoperative period. Vesicles and blisters could be avoided by using cool wraps on a daily basis. In order to prevent potential cutaneous superinfections, it was also requested that an antibiotic ointment, gentamicin 0.1%, be applied to the target areas for 7 days following each laser session. Possible side effects such as blisters, hyper/hypopigmentation and scarring were monitored.

### 2.3 Efficacy and safety assessments

Treatment outcome was assessed by ranking the results into four categories, a quartile scale, of lesion clearance in comparison to baseline: 1 indicates no or low results (0%−25% of the lesion area cleared), 2 indicates slight clearance (25%−50% of the lesion area cleared), 3 indicates moderate‐good clearance (50%−75%), and 4 indicates excellent clearance (75%−100%).

Patients were asked for a subjective evaluation of the perceived overall results by means of the following score: unsatisfied (1), not very satisfied (2), satisfied (3), and very satisfied (4).

Results obtained were judged with a photographic evaluation, immediately and at the control visit (6 months after the last laser session).

## 3 RESULT

For this study, 203 patients with multiple viral warts (51% men and 49% women) with ages ranging from 7 to 61 years (28.3 ± 18.1), were carefully selected. Sixty three of these patients were children aged 7–10 years or over (22% phototype I, 44% phototype II, 34% phototype III).

In particular, lesions were located on the hands (palms, dorsum, and fingertips) and on the feet. Most of these multiple lesions were periungual warts. All patients underwent 4−5 treatment (4.4 ± 0.5) sessions with a 595 nm pulse Dye Laser (Synchro Vas‐Q, Deka M.E.L.A, Calenzano, FI, Italy). Only in cases of typical hyperkeratosis, treatment sessions were preceded by the application of salicylic acid 30% ointment in order to soften the skin and consequently facilitate the application of the laser.

**FIGURE 1:**
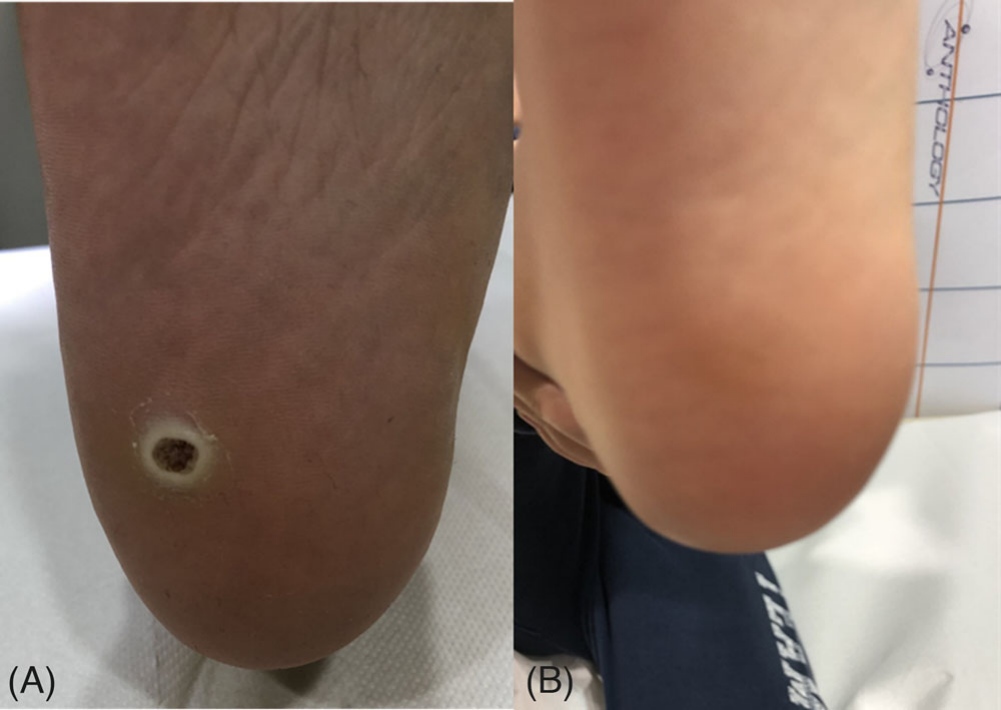
Plantar warts exhibiting full resolution with 595 nm laser treatment. Before treatment (A); Complete resolution at 6 months follow up after the last laser treatment session (B).

**FIGURE 2:**
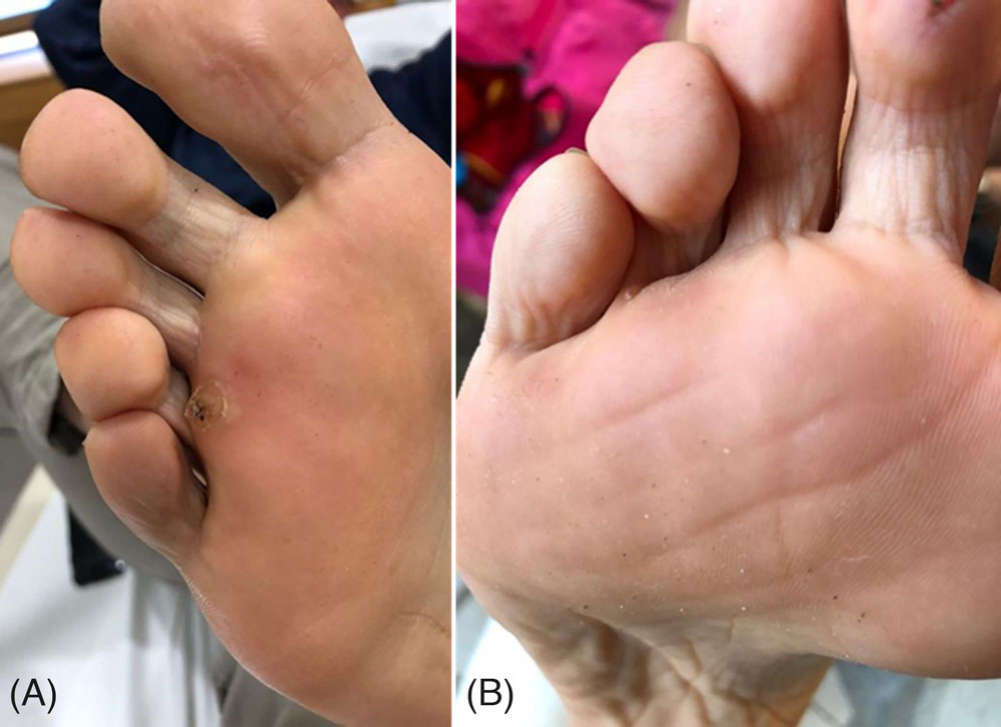
Plantar warts exhibiting full resolution with 595 nm laser treatment. Before treatment (A); Complete resolution at 6 months follow up after the last laser treatment session (B).

**FIGURE 3:**
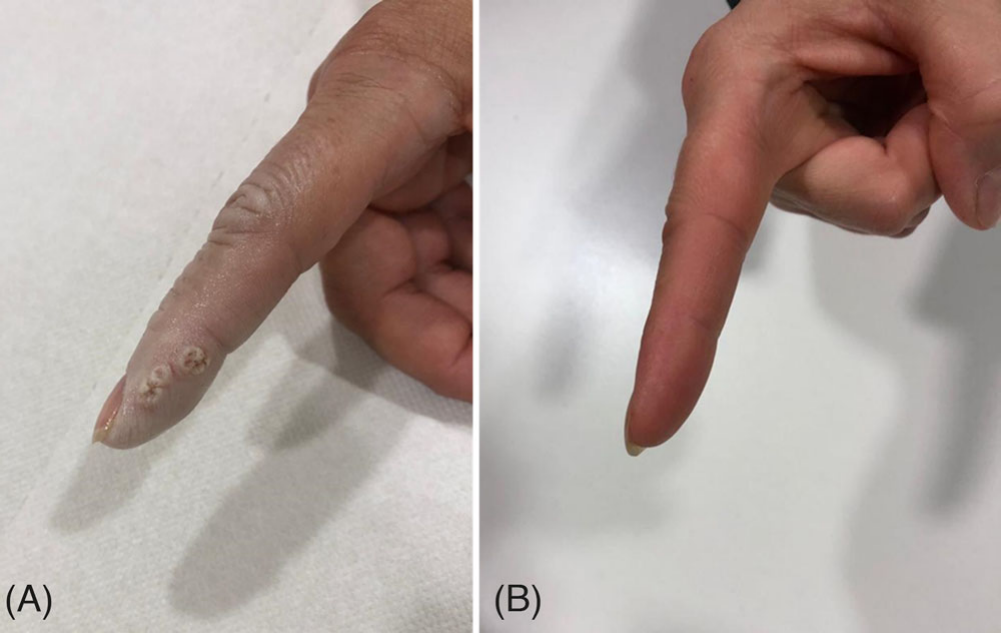
A female patient with periungual hyperkeratosis warts around the finger (A). A substantial improvement at 6 months follow up after the last laser treatment session was observed (B).

**FIGURE 4:**
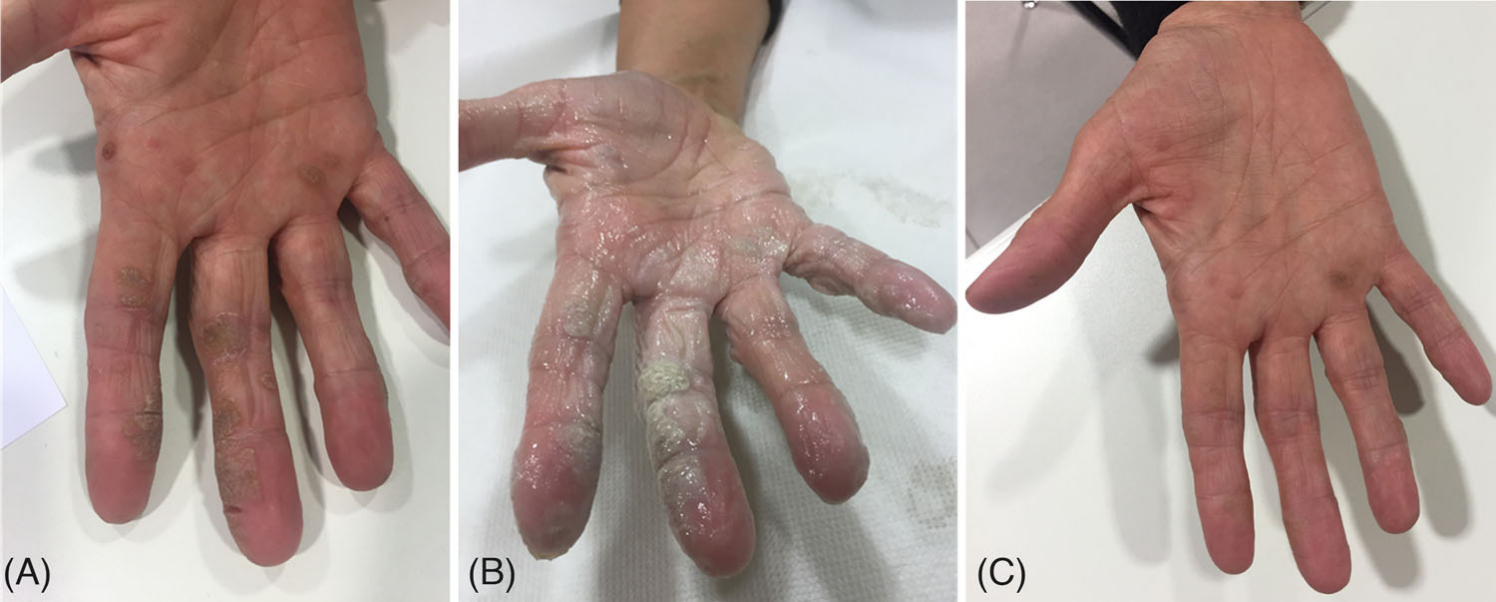
The progress of laser treatment of palmoplantar hyperkeratosis warts in a 60‐year‐old female patient. The wart before laser treatment (A). Palmar wart with salicylic acid ointment before laser treatment (B). Evaluation of the improvement of palmoplantar warts at 6 months follow up (C).

**FIGURE 5:**
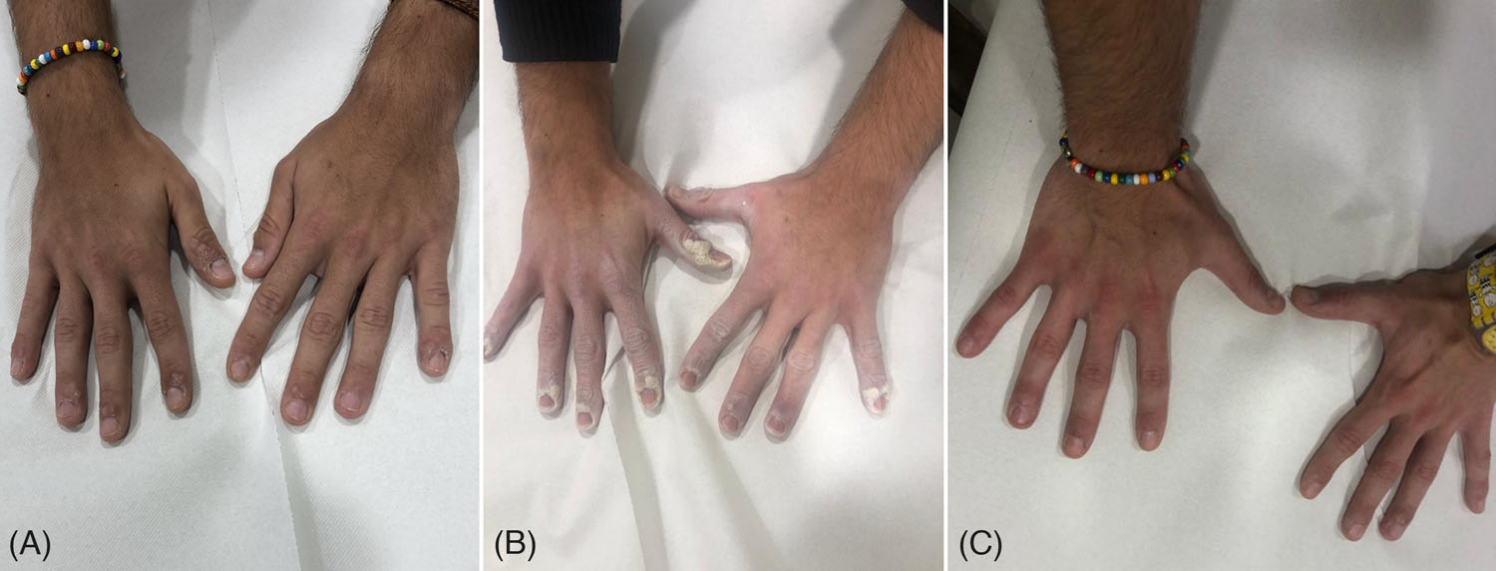
The progress of laser treatment of hyperkeratosis warts on dorsal hands in a male patient. The wart before laser treatment (A). Warts with salicylic acid ointment before laser treatment (B). A substantial improvement at 6 months follow up after the last laser treatment session was observed (C).

All patients observed global improvements. Most of the lesions were completely removed after laser therapy. Total of 95% of patients achieved excellent clearance and they were very satisfied following the laser treatment. Physician evaluation revealed 91% excellent clearance and 9% moderate‐good clearance. The assessment of patient satisfaction was 95% very satisfied and 5% very satisfied. Post‐operative time is only characterized by bruises and healing occurs without scarring. Relevant side effects, such as blisters, crusts, atrophy, and scars, were absent in all patients. Some clinical cases with a visible improvement and a complete resolution of the viral warts, after 595 nm laser treatment, are shown in Figures 1, 2, 3, 4, 5.

## 4 DISCUSSION

A combination of lasers with keratolytic agents, immunomodulators, and photodynamic therapy can be helpful, especially in immunosuppressed patients, refractory, and recurrent lesions. PDL has the lowest occurrence of adverse effects relative to other types of lasers.^16^

PDL functions by impairing the blood supply, and thereby oxygen and nutrients, to proliferating keratinocytes infected by viral particles. The treatment is less time‐consuming compared to other treatments, such as electrosurgery and CO_2_ treatments. Local anesthesia is often unnecessary.^17^

The mechanism of action of PDL is thereby based on the specific destruction of abnormal vessels, components of the lesions themselves (angiokeratoma circumscriptum and striae rubrae), or a selective thrombosis of vessels with the consequent obliteration of the nutrient supply to the lesions (viral wart, angiolymphoid hyperplasia, Kaposi's sarcoma, and basal cell carcinoma). Specifically, the damage of virally infected keratinocytes by PDL may contribute to the treatment of warts because the human papillomavirus is heat‐sensitive.^18^, ^19^

The paring of the keratotic component of warts with a blade, to the extent that blood vessels are seen without causing bleeding before PDL treatment, is thought to provide deeper penetration of the laser light.^20^

The study evidences an overall patient improvement that may represent a valid alternative to other procedures.

Moreover, although it has been demonstrated in the literature that, the long‐pulsed Nd:YAG laser was used for the treatment of HPV lesions,^21^ and that it was also able to induce a selective photothermolysis for blood vessels,^22^ some studies demonstrated that there is no significant difference in the effectiveness between both laser types^23^; the study of Ibrahim and colleagues showed that PDL and Nd:YAG laser are effective and safe modalities in recalcitrant plantar warts treatment with tolerable side effects.^24^ Specifically, CO_2_, PDL, and Nd:YAG are the laser modalities most studied for wart treatment, and of these, PDL has the most favorable adverse effect profile.^16^, ^25^

On these bases, we selected PDL therapy for this study. Our results showed that 595 nm pulse dye‐laser was highly effective for inducing the clearance of the lesions.

Evaluation of the treatment results at 6 months follow‐up after the initial treatment showed complete clearance of the warts in 95% of the patients. All patients expressed a high degree of satisfaction following the laser treatment, and most of them did not return for further sessions.

Moreover, the rate of recurrence was negligible, seemingly lower than other therapeutic interventions.

In this study, the use of salicylic acid ointment was limited to only cases of hyperkeratotic warts in order to facilitate the application of the dye‐laser at the beginning and between treatment sessions.

Compared to ablative treatments, the post‐operative time is only characterized by bruises and healing occurs without scarring. Indeed, in comparison to other invasive techniques, dye laser does not generate scars, the procedure can be repeated as many times as desired, and it does not generate the “incarceration” of viral lesions, which could render it resistant. In addition, this laser technique, does not expose to de‐epithelialization due to external agents.

No scarring, post‐hyperpigmentary changes or serious adverse events were documented. Our preliminary results show that 595 nm laser treatments are safe and effective for warts of hands and feet, causing minimal discomfort, and is a viable treatment alternative.

In conclusion, we recommend this therapeutic approach above all to treat multiple warts and periungual areas.

Future studies to examine the optimal laser parameters and treatment intervals may be conducted to enhance our knowledge of how best to use for successful management of warts.

## 5 CONCLUSIONS

PDL treatment with the study device using a wavelength of 595 nm has proven to be a tolerable and safe therapy for the viral warts management.

## CONFLICT OF INTEREST STATEMENT

Irene Fusco, Lara Ronconi, and Tiziano Zingoni are employed at El.En. Group. The other authors declare that the research was conducted in the absence of any commercial or financial relationships that could be construed as a potential conflict of interest.

## INSTITUTIONAL REVIEW BOARD STATEMENT

The study was conducted in accordance with the Declaration of Helsinki. Ethics review and approval for this study were waived because device has already been CE‐marked since 2013.

## INFORMED CONSENT STATEMENT

All participants in the study provided their informed consent.

## Data Availability

On reasonable request, the corresponding author will provide the information supporting the study's results.
